# Prevalence of SARS-COV-2 and other respiratory pathogens among a Jordanian subpopulation during Delta-to-Omicron transition: Winter 2021/2022

**DOI:** 10.1371/journal.pone.0283804

**Published:** 2023-03-30

**Authors:** Ashraf I. Khasawneh, Nisreen M. Himsawi, Jumana A. Abu-Raideh, Ashraf Sammour, Hazem Abu Safieh, Ali Obeidat, Mohammed Azab, Amjed A. Tarifi, Abdallah Al Khawaldeh, Hafez Al-Momani, Sofian Al Shboul, Tareq Saleh

**Affiliations:** 1 Department of Microbiology, Pathology, and Forensic Medicine, Faculty of Medicine, The Hashemite University, Zarqa, Jordan; 2 Department of Anatomy, Physiology, and Biochemistry, Faculty of Medicine, The Hashemite University, Zarqa, Jordan; 3 Department of Otorhinolaryngology, Irbid Specialty Hospital, Irbid, Jordan; 4 Department of Specialized Surgery, Faculty of Medicine, The Hashemite University, Zarqa, Jordan; 5 Department of Pharmacology and Public Health, Faculty of Medicine, The Hashemite University, Zarqa, Jordan; Children’s National Hospital, George Washington University, UNITED STATES

## Abstract

Acute respiratory tract infections (ARTIs) during the winter months are associated with higher morbidity and mortality compared to other seasons of the year, with children below five, elderly, and immunocompromised patients being the most susceptible. Influenza A and B viruses, rhinovirus, coronaviruses, respiratory syncytial virus, adenovirus, and parainfluenza viruses, are the most frequently identified causes of viral ARTIs. In addition, the emergence of SARS-CoV-2 in 2019 provided an additional viral cause of ARTIs. The aim of this study was to provide an overview of the epidemiological status of upper respiratory infections, their main causative agents, and reported clinical presentation in the winter months of 2021, during two important surges of COVID-19 in Jordan. Nasopharyngeal samples were collected from 339 symptomatic patients during the period from December 2021 to March 2022, followed by nucleic acid isolation using a Viral RNA/DNA extraction Kit. The causative virus species associated with the patient’s respiratory symptoms was determined utilizing a multiplex real-time PCR targeting 21 viruses, 11 bacteria, and a single fungus. SARS-CoV-2 was identified in 39.2% of the patients (n = 133/339). A total of 15 different pathogens were also identified as co-infections among these 133 patients (n = 67/133). SARS-CoV-2-Bacterial coinfections (37.6%, n = 50/133) were the most frequent, with *Bordetella* species being the most common, followed by *Staphylococcus aureus*, and *H*.*influenzae* type *B*. Viral coinfection rate was 27.8% (n = 37/133), with Influenza B virus and Human bocavirus being the most common. In Conclusion, Both SARS-CoV-2, influenza B virus, and *Bordetella* accounted for the majority of infections in patients with URTI during the winter months of 2021–2022. Interestingly, more than 50% of the patients with symptoms of URTIs were confirmed to have a coinfection with two or more respiratory pathogens, with SARS-CoV-2 and *Bordetella* coinfection being most predominant.

## Introduction

Acute respiratory tract infection (ARTI) during the winter is linked to higher morbidity and mortality in kids and adults compared to other times of the year. Children under the age of five years, people with compromised immune systems, and the elderly are the most vulnerable populations [1, 2]. The main pathogens implicated in ARTIs are viruses. Influenza A and B viruses, rhinoviruses, coronaviruses, respiratory syncytial viruses (RSV), adenoviruses, and parainfluenza viruses are the most common viruses circulating in the community and strongly associated with ARTIs in the general population. However, their incidence and distribution differ by geographic region, season, and age group [3, 4]. The clinical picture observed in patients with ARTI regardless of the causative microorganism is largely similar, and thus, the precise diagnosis of ARTIs relies almost entirely on laboratory investigation. ARTIs patients present mostly with fever, runny nose, sore throat, cough, sneezing, and myalgia and less frequently chest tightness [5].

In December 2019 a new coronavirus, severe acute respiratory coronavirus 2 (SARS-CoV-2), emerged in Wuhan, China [6]. COVID-19 pandemic started with α, β, and γ SARS-CoV-2 variants which were predominant and of concern in December 2020 [7]. Infection with these variants was associated with lower respiratory tract infection and increased mortality [8]. Around the turn of the year the omicron variant emerged overtaking the Delta variant as the dominant strain [9]. The omicron variant started as the BA.1 subvariant and continued to mutate until the emergence of the new omicron subvariants BA.4 and BA.5 which were more stable and not associated with severe illness compared to their earlier counterparts [10]. Despite being more contagious, BA.5, is associated with influenza like symptoms [11]. Early during the pandemic differentiating SARS-CoV-2 from influenza was of utmost importance since their treatment protocols were different. Over 2021 and 2022, subvariant BA.5 has become the most dominant strain and the clinical picture of SARS-CoV-2 infection tended to manifest as upper respiratory tract infection (URTI) that is treated like common cold or flu.

Each year during the winter months an epidemic of influenza and common cold viruses such as rhinoviruses, coronaviruses (229E, OC43, NL63 and HKU1), adenoviruses, and the human metapneumovirus occur [12]. Jordan has witnessed an increase in the number and severity of H1N1 influenza virus infections in 2019 winter, in addition to the usual common cold viral infections [13]. During the last winter (2021) and with the local and global surge in COVID-19 cases it was important to investigate the epidemiological status of the viruses which will be circulating in the population to determine the efficacy of the COVID-19 preventive measures, help health policy makers in setting up the appropriate response measures, and set early alarms to health officials should big foci of COVID-19 and/or H1N1 spread in the central and northern parts of Jordan. Despite continuing to offer free COVID-19 testing for the general population, daily or weekly COVID-19 statistics are no longer publicly available, making several healthcare facilities, especially in the private sector, not fully updated with accurate data regarding the continuous surges of URTIs caused by COVID-19. One of the aims of this study was to offer a glance on the expected surge in the coming winter and provide an overview of the respiratory pathogens including SARS-CoV-2. The early detection of causative pathogens is critical to limit the spread of infection, provide the best tailored treatment regimen, and shorten the overall duration of hospital stay [14, 15]. Currently, ARTIs causative pathogens can be determined by multiple different laboratory diagnostic methods such as the utilization of conventional viral cell cultures and antigen detection tests. Cell culture remains the gold-standard technique for virus detection, but it is associated with inaccurate results in the early phase of disease in addition to being an arduous and lengthy process [16]. Serological tests are more rapid but can be associated with subordinate sensitivity and/or specificity [17, 18]. On the other hand, the currently available molecular diagnostic methods represent a more popular tool for faster and more accurate diagnosis and are associated with excellent sensitivity and specificity. Multiplex real-time polymerase chain reaction (RT-PCR) assays provide a single-step amplification of numerous viruses or bacteria in one reaction making it easier to detect the most common ARTIs causative agents [19]. Furthermore, new testing kits allow for the detection of newly emerging respiratory viruses, such as seasonal HCoV (229E, OC43, NL63, HKU1), MERS, SARS, SARS-CoV-2, and human metapneumovirus (HMPV), which are difficult to propagate in the cell culture [20, 21].

The aim of this study was to provide an overview of the epidemiological status of URTI, their main causative agents, and reported clinical presentation in the winter months of 2021, during two important surges of COVID-19 in Jordan. For that, nasopharyngeal specimens were collected from symptomatic patients, genetic material extracted, and real-time PCR testing was performed to identify the causative pathogens in three major cities in Central and Northern Jordan.

## Materials and methods

### Study population

This study was conducted in three hospitals in three densely populated cities in Jordan, namely Prince Hamza Hospital (PHH) in Amman, Irbid Specialty Hospital (ISH) in Irbid, and Jabal Al-Zaytoun Hospital (JZH) in Zarqa from December 2021 to March 2022. Patients seeking medical attention at the emergency department (ED) with symptoms of URTIs were recruited in this study. Symptoms included: fever, sore throat, runny nose, sneezing, cough, and general fatigue. Patients with confirmed COVID-19 status and children below five years were excluded from this study. Samples were obtained using a nasopharyngeal flocked swab (ZYBIO, China), then transferred into sterile tubes containing 3 ml of cryoprotectant solution where samples were processed directly or stored at -20°C for Nucleic acid extraction. All CDC recommendations regarding proper and safety aseptic techniques for sample collection and transportation protocols were taken into consideration all over the study.

This study obtained the Institutional Review Board (IRB) approvals from The Hashemite University (No. 3/7/2020/2021) and PHH (No. MH/ 517/2022) according to the established guidelines. All enrolled participants or guardians gave a written informed consent prior to participation in this study. Patient demographics, clinical picture, health status, vaccine status, and COVID-19 exposure history data were collected using a standard questionnaire.

### Nucleic acid extraction and SARS-CoV-2 detection

Combined oropharyngeal/nasal swabs were collected from 339 patients and transported to the Virology Research Laboratory at the Faculty of Medicine in the Hashemite University. The total nucleic acid was isolated from the nasopharyngeal swabs using the QIAamp MinElute Virus Spin Kit (Qiagen, Germany) according to the manufacturer’s instructions. Nucleic acid was eluted in 200 μl of AE buffer and stored at -20°C for subsequent processing. Nucleic acid concentration was measured using Qubit 3.0 Fluorometer (Thermo Fisher Scientific, USA) as detailed by the manufacturer. To detect SARS-CoV-2, the ZYBIO SARS-CoV-2 detection kit was used according to manufacturer’s instructions (ZYBIO, China). The kit designed with self-reverse transcription step in order to generate the complementary DNA (cDNA) in the next step the resulting cDNA combines with specific primers and probes referring to the conserved sequences of N, RdRP and E genes of SARS-CoV-2. The RT-PCR assays were conducted on Bioer Real-Time PCR System according to the manufacturer’s instructions. The threshold cycle (Ct) value was assigned for each PCR run, and the amplification curves were assessed visually. Assessment of specimens’ results were performed after the positive and no template (negative) controls have been examined and determined to be valid and acceptable in each run.

### FTD respiratory pathogens 33 multiplex panel

To determine the causative viral species associated with the patient’s respiratory symptoms, other than SARS-CoV-2, we performed a multiplex RT-PCR runs targeting 21 viruses, 11 bacteria, and a single fungus, using the FTD Resp33 Panel (Fast-track Diagnostics, Germany). The FTD assay is a one-step RT-PCR containing primer-probe mixtures for the simultaneous amplification of 33 respiratory pathogens: influenza A virus (IAV), influenza A swine virus(H1N1), influenza B virus (IBV), influenza C virus (IVC), human coronaviruses (HCoVs) including NL63, 229E, OC43, and HKU1 types, human parainfluenza viruses (HPIVs) 1, 2, 3, and 4, human metapneumoviruses (HMPVs) A and B, human rhinovirus (HRV), human respiratory syncytial viruses (HRSVs) A and B, human adenovirus (HAdV), enterovirus (EV), human parechovirus (HPeV), human bocavirus (HBoV), *Pneumocystis jirovecii*, *Mycoplasma*. *pneumoniae*, *Chlamydophila pneumoniae*, *Streptococcus pneumoniae*, *Haemophilus influenzae* type b, *Staphylococcus aureus*, *Moraxella catarrhalis*, *Bordetella* spp., *Klebsiella pneumoniae*, *Legionella pneumophila* and *Legionella*. *longbeachae*, *Salmonella* spp., *Haemophilus influenzae* (non-type b), and equine arteritis virus (EAV), which was included as an internal control (IC).

Eight independent multiplex RT-PCR reactions were set up following the manufacturer’s instructions. Each PCR reaction was carried out in a 25 μl final volume where 10 μl of the extracted nucleic acid samples were mixed with 15 μl of the kits’ master mix. The multiplex real-time RT-PCR thermal profile was as the following: 50°C for 15 minutes, 94°C for 1 minute, 40 cycles of 94°C for 8 seconds, and 60°C for 1 minute. A sample was considered positive for a microorganism where a sigmoidal curve within a cycle threshold (*C*_*T*_) value of < 40 was recorded. The IC was extracted with the specimens and used with each PCR run along with positive and negative controls provided by the manufacturer.

### Statistical analysis

Statistical analysis was performed using IBM SPSS (Statistical Package for the Social Sciences) version 24 with the exact test package (IBM, USA). Patient variables were analyzed using the descriptive statistics analysis for frequencies and ratios, the differences between the study groups were tested by Pearson Chi-Square test. *p* values equal to or less than 0.05 were considered statistically significant.

## Results

### Patients’ characteristics

Three hundred and thirty-nine patients presented with URTIs symptoms, the three most common symptoms were headache, sore throat, and cough which were seen in 75.2%, 74.6%, and 74.0%, respectively (**Table 1**). Myalgia, nasal discharge and congestion, fever and chills were seen in more than 50% of the patients. On the other hand, gastrointestinal disturbances were reported less frequently (7.1–19.2%) (**Table 1**). Chills and nasal discharge symptoms were statistically significant presenting symptoms in SARS-CoV-2 infected patients, likewise, myalgia, cough, and nasal discharge were statistically significant presenting symptoms in patients infected with IBV (**S1 Table**). The mean age ± SD was 32.77 ± 15.8 years, ranging from 5 to 70 years old, with most patients in the age range of 19–65 years (68.7%), and males accounted for 59.3% of the participants (**Table 1**). Most cases of SARS-CoV-2 infections were seen among the patients aged between 11–65 years old (*p* = 0.021), while most IAV infections were seen among patients aged between 19–45 years old with a statistically significant difference (*p* = 0.001), also male predominance was seen among patients infected with IAV (*p* = 0.001) (**S2 Table**). One hundred and eighty-seven (55.2%) out of 339 samples were collected at Prince Hamza Hospital (PHH). Furthermore, most participants had no pre-existing disease (79.4%), while participants with chronic illnesses and pre-existing respiratory conditions represented 9.4% and 8.0%, respectively.

**Table 1:** Demographic and clinical status characteristics of study participants.

Variable	N = 339	%
**Gender**		
Male	201	59.3
Female	138	40.7
**Age**		
5–10	2	0.6
11–18	69	20.4
19–45	116	34.2
46–65	117	34.5
> 65	35	10.3
**Symptoms during sample collection**		
Headache	255	75.2
Sore throat	253	74.6
Cough	251	74.0
Myalgia	227	67.0
Nasal discharge and congestion	202	59.6
Chills	199	58.7
Persistent fever	175	51.6
Difficulty in breathing	117	34.5
Dizziness	101	29.8
Vomiting	65	19.2
Nausea	65	19.2
Change of smell or taste	38	11.2
Diarrhea	24	7.1
**Health status**		
Healthy	269	79.4
Chronic diseases (Diabetes, Hypertension, etc.)	32	9.4
Respiratory diseases (Sinusitis, Frequent tonsillitis Seasonal allergy, Asthma)	27	8.0
Other diseases	11	3.2

Most participants (99.7%) reported no previous hospital admission in the past 14 days before the emergence of their current symptoms. Additionally, almost two-thirds (62.8%) of the participants did not recall any close contact with a confirmed COVID-19 patient; however, the remaining one-third reported a close contact with family members and/or co-workers who were COVID-19 positive (**Table 2**). The majority of the participants in this study (69.8%) received two doses of the SARS-CoV-2 vaccine of which Pfizer-Biontech mRNA vaccine and Sinopharm were taken by 39.2% and 32.2% of the study participants, respectively (**Table 2**). SARS-CoV-2 vaccine has shown a protective effect against infection or reinfection with SARS-CoV-2 among the studied population (p = 0.046) (S2 Table).

**Table 2:** Participants response regarding COVID-19 infection and SARS-COV-2 vaccination status.

Question	Answer	N = 339	%
**Were you admitted to the hospital in the past 14 days?**	Yes	1	0.3
	No	338	99.7
**During the past 5 days, have you been in close contact with a confirmed COVID-19 case?**	Yes (Work)	49	14.5
	Yes (Family)	76	22.4
	Yes (Hospital)	1	0.3
	No	213	62.8
**Were you tested for COVID-19 in the past 14 days?**	Yes	80	23.6
	No	259	76.4
**The result of the last COVID-19 test was?**	Negative	64	18.9
	Positive	0	0.0
	Waiting for result	14	4.1
	NA	261	77.0
**Are you fully/partially vaccinated?**	First dose	7	2.1
	Second dose	237	69.9
	Third dose	24	7.1
	Unvaccinated	71	20.9
**Type of SARS-CoV-2 vaccine did you receive?**	Pfizer-Biontech	133	39.2
	Sinopharm	109	32.2
	AstraZenca	17	5.0
	Sputnik	2	0.6
	Mixed (Pfizer & Sinopharm)	7	2.1
	Unvaccinated	71	20.9

### Infections and coinfections with SARS-CoV-2

**Fig 1:**
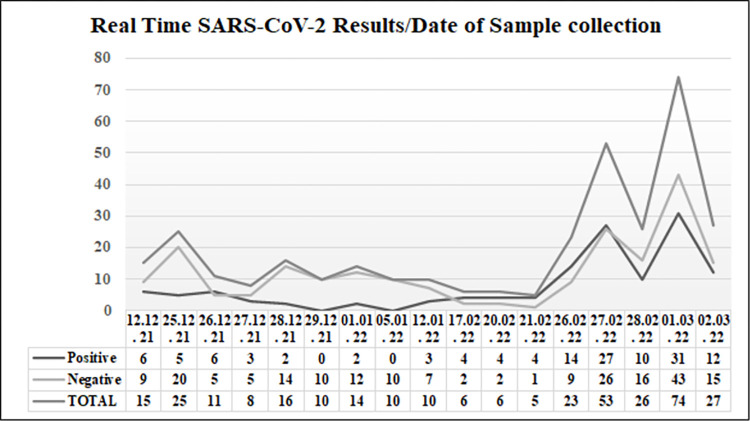
Temporal distribution for SARS-COV-2 testing results over the study period.

During the study period, all of the 339 patients with URTIs were tested for SARS-CoV-2 infection. The pattern of SARS-CoV-2 testing and infection over the study period is shown in (**Fig 1**). SARS-CoV-2 was recorded in 133 (39.2%) patients (**Table 3**). Among these 133 patients, a total of 67 (50.4%) had other viral, or bacterial coinfection identified by the FTD-33 assay (**Table 4**). Around one quarter (n = 37, 27.8%) of the 133 patients had other respiratory viral coinfections (viral only, n = 17, or viral-bacterial, n = 20), and 50 (37.6%) had bacterial coinfections (bacterial only, n = 30, or bacterial-viral, n = 20). None of the patients had fungal coinfection with *Pneumocystis jirovecii* (**Table 3**). Specifically, a single virus (other than SARS-CoV-2) was detected in 17 samples, and a single bacterium was identified in 26 samples in patients with SARS-CoV-2 (**Table 5**). Multiple co-detections were present in 24 samples, including 20 double detections (mixed bacterial, n = 3; mixed viral, n = 2, and bacterial-viral, n = 15), 3 triple detections (mixed bacterial, n = 1; bacterial-viral, n = 2), and 1 quadruple detection (bacterial-viral) (**Table 4**).

**Table 3:** Respiratory-pathogen frequencies identified via the fast track diagnostics real-time PCR kit (N = 339).

Pathogen Identified	Positive N (%)	Negative N (%)
SARS-CoV-2	133 (39.2)	206 (60.8)
FTD (All pathogens)	194 (57.2)	145 (42.8)
Any respiratory pathogen (SARS-CoV-2 and FTD)	258 (76.1)	81 (23.9)
Influenza A virus **(IAV)**	37 (10.9)	302 (89.1)
Influenza B virus **(IBV)**	59 (17.4)	280 (82.6)
Influenza C virus **(ICV)**	4 (1.2)	335 (98.8)
Influenza A (H1N1) virus **IAV(HINI)**	0	339 (100)
Human Parainfluenza viruse-1 **(HPIV-1)**	0	339 (100)
Human Parainfluenza viruse-2 **(HPIV-2)**	1 (0.3)	338 (99.7)
Human Parainfluenza viruse-3 **(HPIV-3)**	1 (0.3)	338 (99.7)
Human Parainfluenza viruse-4 **(HPIV-4)**	0	339 (100)
Human coronavirus NL63 **(HCoV NL63)**	0	339 (100)
Human coronavirus 229E **(HCoV 229E)**	6 (1.8)	333 (98.2)
Human coronavirus OC43 **(HCoV OC43)**	0	339 (100)
Human coronavirus HKU1 **(HCoV HKU1)**	2 (0.6)	337 (99.4)
Human metapneumovirus A and B **(HMPV A &B)**	0	339 (100)
Human rhinovirus **(HRV)**	2 (0.6)	337 (99.4)
Human respiratory syncytial viruses A and B **(HRSV A&B)**	3 (0.9)	336 (99.1)
Human adenovirus **(HAdV)**	3 (0.9)	336 (99.1)
Enterovirus **(EV)**	16 (4.7)	323 (95.3)
Human parechovirus **(HPeV)**	4 (1.2)	335 (98.8)
Human bocavirus **(HBoV)**	16 (4.7)	323 (95.3)
*Pneumocystis jirovecii fungus* ***(P*.*jirovecii)***	0	339 (100)
*Mycoplasma pneumoniae* ***(M*. *pneumoniae)***	0	339 (100)
*Chlamydophila pneumoniae* ***(C*. *pneumoniae)***	0	339 (100)
*Streptococcus pneumoniae* ***(S*. *pneumoniae)***	9 (2.7)	330 (97.3)
*Haemophilus influenzae type B* ***(H*. *influenzae B)***	32 (9.4)	307 (90.6)
*Staphylococcus aureus* ***(S*. *aureus)***	40 (11.8)	299 (88.2)
*Moraxella catarrhalis* ***(M*. *catarrhalis)***	10 (2.9)	329 (97.1)
*Bordetella spp*. ***(all Bordetella except Bordetella parapertussis)***	59 (17.4)	280 (82.6)
*Klebsiella pneumoniae* ***(K*. *pneumoniae)***	0	339 (100)
*Legionella pneumophila/ Legionella longbeachae* ***(L*. *pneumophila /L*. *longbeachae)***	1 (0.3)	338 (99.7)
*Salmonella spp*.	0	339 (100)
*Haemophilus influenzae* ***(H*. *influenzae)***	8 (2.4)	331 (97.6)

**Table 4:** List of single pathogen infections and pathogen-coinfections identified via FTD assay among all participants.

*No*. *of patients’ cases*						
Pathogen	Single pathogen	Single coinfection	Double coinfection	Triple coinfection	More than triple coinfection	Total
**SARS-Cov-2 (n = 133)**	66	43	20	3	1	133
**IAV (n = 37)**	14	12	7	3	1	37
**IBV (n = 59)**	18	23	13	3	2	59
**ICV (n = 4)**		2		1	1	4
**HPIV-2 (n = 1)**				1		1
**HPIV-3 (n = 1)**	1					1
**HCoV 229E (n = 6)**	2	1	3			6
**HCoV HKU1 (n = 2)**		1	1			2
**HRV (n = 2)**		1	1			2
**HRSV A&B (n = 3)**	1	1			1	3
**HAdV (n = 3)**		2	1			3
**EV (n = 16)**		6	7	3		16
**HPeV (n = 4)**		1	2		1	4
**HBoV (n = 16)**	1	6	6	3		16
***S*. *pneumoniae* (n = 9)**	1	4	4			9
***H*. *influenzae B* (n = 32)**	3	8	10	9	2	32
***S*. *aureus* (n = 40)**	13	13	9	2	3	40
***M*. *catarrhalis (*n = 10)**	3	1	5	1		10
***Bordetella spp* (n = 59)**	7	27	16	7	2	59
***L*. *pneumophila /L*. *longbeachae* (n = 1)**					1	1
***H*. *influenzae* (n = 8)**	3	2	3			8

**Table 5:** List of singular coinfections (microorganism- microorganism) that were identified among the study participants.

Pathogen	SARS-CoV-2	IAV	IBV	ICV	HCoV 229E	HCoV HKU1	HRV	HRSV A&B	HAdV	EV	HPeV	HBoV	*S*. *pneumoniae*	*H*. *influenzae B*	*S*. *aureus*	*M*. *catarrhalis*	*Bordetella spp*.	*H*. *influenzae*
**SARS-CoV-2 (n = 43)**	**NA**		**9**						**1**	**3**		**4**	**1**	**2**	**7**	**1**	**14**	**1**
**IAV (n = 11)**		**NA**	**3**		**1**								**2**		**1**		**4**	
**IBV (n = 23)**	**9**	**3**	**NA**	**1**			**1**			**1**	**1**	**1**		**2**	**2**		**1**	**1**
**ICV (n = 2)**			**1**	**NA**													**1**	
**HCoV 229E (n = 1)**		**1**			**NA**													
**HCoV HKU1 (n = 1)**						**NA**									**1**			
**HRV (n = 1)**			**1**				**NA**											
**HRSV A&B (n = 1)**								**NA**							**1**			
**HAdV (n = 2)**	**1**								**NA**			**1**						
**EV (n = 6)**	**3**		**1**							**NA**							**2**	
**HPeV (n = 1)**			**1**								**NA**							
**HBoV (n = 6)**	**4**		**1**						**1**			**NA**						
***S*. *pneumoniae* (n = 4)**	**1**	**2**											**NA**				**1**	
***H*. *influenzae B* (n = 8)**	**2**		**2**											**NA**	**1**		**3**	
***S*. *aureus* (n = 13)**	**7**	**1**	**2**			**1**		**1**						**1**	**NA**			
***M*. *catarrhalis (*n = 1)**	**1**															**NA**		
***Bordetella spp*. (n = 26)**	**14**	**4**	**1**	**1**						**2**			**1**	**3**			**NA**	
***H*. *influenzae*(n = 2)**	**1**		**1**															**NA**

**Fig 2:**
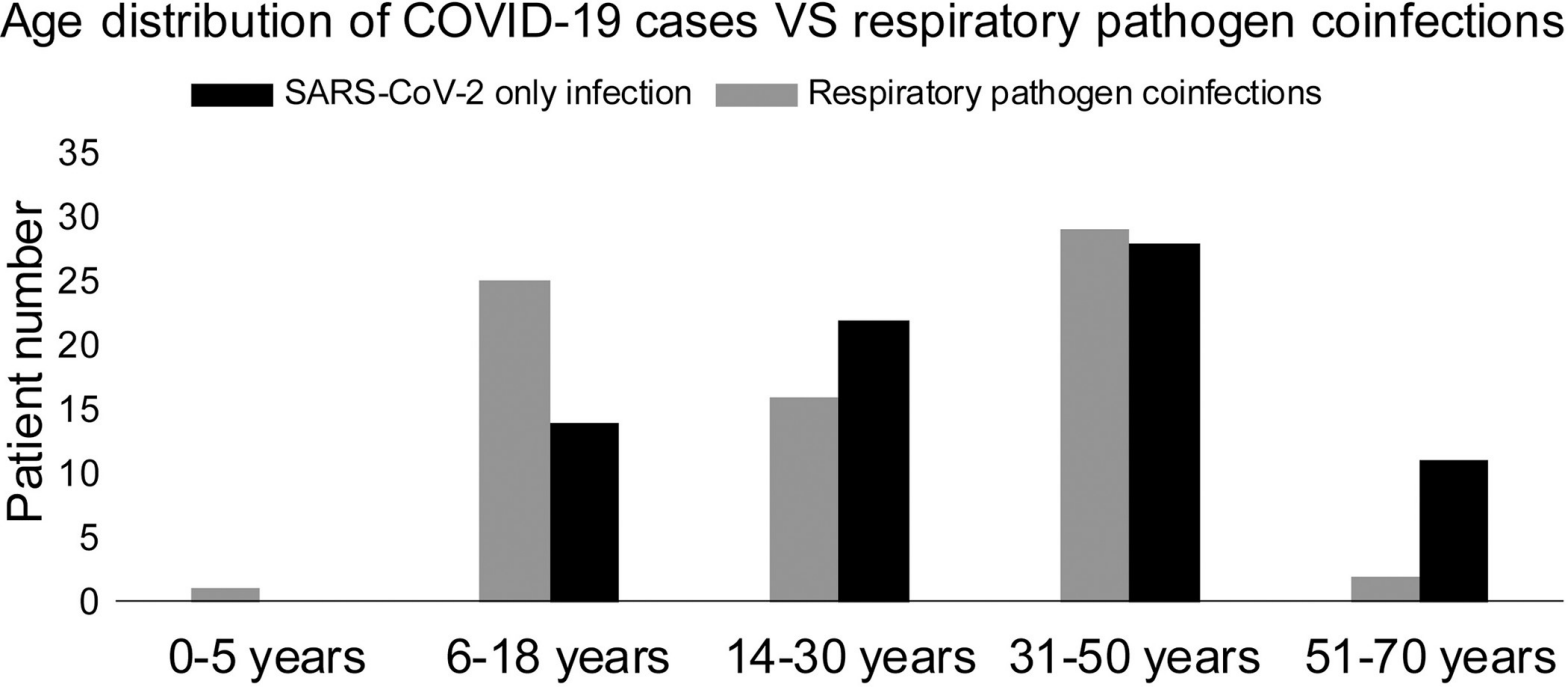
Age distribution of SARS-CoV-2 only infection (n = 66) vs. COVID-19 cases with respiratory pathogen coinfection (n = 67) among the study population.

The male to female ratio in the coinfection cases was 1.46:1, a statistically significant difference in coinfection by gender was not observed (*p* = 0.48), suggesting that gender difference played no role in susceptibility to coinfection with other respiratory pathogens among SARS-COV-2 positive patients. The mean age of the coinfection cases (n = 67) with other respiratory pathogens (a virus or a bacteria) was 27.4 ± 14.6 years, which was younger but not statistically significant (*p* = 0.21) compared to the mean age of patients without coinfections which was 32.8 ± 15.9 years. Most of the respiratory pathogen coinfection cases were detected in the Capital Province (n = 46, 24.9% of the study population at Amman), followed by Irbid Province (n = 18, 30.5% of the study population at Irbid) and Al Zarqa Province (n = 3, 3.4% of the study population at Al Zarqa). **Fig 2** shows the age distribution of respiratory pathogen coinfection cases (n = 67) and SARS-CoV-2 only infection (n = 66) among the study population (n = 339).

### Infections and coinfections with other respiratory viruses and bacteria

Among our study participants’ URTIs were caused by multiple viruses other than SARS-CoV-2, as influenza virus was seen to account for most URTIs [IBV n = (59, 17.4%); IAV (n = 37, 10.9%) and ICV (n = 4, 1.2%)], followed by Human bocavirus (HBoV) n = 16 (4.7%), and enterovirus (EV) n = 16 (4.7%) (**Table 3**). Non-SARS-CoV-2 coronaviruses (229E and HUK1) were also seen in n = 8 (2.4%) cases. Moreover, among COVID-19 negative patients, IBV caused the most single viral infections (n = 19, 9.2%), followed by IAV (n = 14, 6.8%), HCoV 229E (n = 2, 1.0%), and a solitary case of each of the following viruses: HBoV, HPIV 3, and HRSV A and B (0.5% each) (Data not shown). Single viral infections and viral-viral or viral bacterial coinfections are further detailed in (**Tables 4 and 5**).

Furthermore, multiple bacteria were seen to cause URTIs solely or in combination with other viruses or bacterial strains; *Bordetella* species, *S*. *aureus*, *and H*. *influenzae* type B were the most common causative pathogens at a frequency of n = 59 (17.4%), n = 40 (11.8%), and n = 32 (9.4%) respectively. *S*. *aureus* was the sole cause of infection in 13 cases (6.3%) among COVID-19 negative patients, followed by *Bordetella* species (7, 3.4%), non-typeable *H*. *influenzae* (3, 1.5%), 3 cases each caused by *H*. *influenzae type B* and *M*. *catarrhalis* (1.5% each), and finally 1 case of *S*. *pneumoniae* (0.5%) (**Tables 4 and 5**). Pathogen combinations identified among all single coinfections (n = 68) are illustrated in (**Table 5**).

IAV infection was seen mostly (n = 36, 17.6%) among COVID-19 negative patients which was significantly higher than just a single case in COVID-19 infected patients (*p* = 0.001). Likewise, IBV difference between COVID-19 negative (n = 43) and positive (n = 15) cases was statistically significant (*p* = 0.019) (**Table 6**). Infection with HCoV 229E was seen only among COVID-19 negative patients and showed a significant difference (*p* = 0.046) (**Table 6**). Conversely, none of the bacterial infections among COVID-19 negative and positive patients showed statistically significant difference.

**Table 6:** Comparison among study participant groups test results regarding both SARS-CoV-2 and FTD pathogens coinfection using Pearson X^2^ test analysis.

Characteristic	Participants with Negative COVID-19 result	Participants with Positive COVID-19 result		p-value
	N = 206 (% among the group)	N = 133 (% among the group)		
FTD		122 (59.2)	72 (54.1)	0.589
**IAV**		**36 (17.5)**	**1 (0.7)**	**0.001**
**IBV**		**44 (21.4)**	**15 (11.2)**	**0.019**
ICV		2(1.0)	2(1.5)	0.667
**HCoV 229E**		**6 (2.9)**	**0**	**0.046**
HCoV HKU1		1 (0.5)	1 (0.7)	0.761
HRV		1 (0.5)	1 (0.7)	0.761
HPIV-2		1 (0.5)	0	0.418
HPIV-3		1 (0.5)	0	0.418
HBoV		7 (3.4)	9 (6.7)	0.161
HRSV A&B		3 (1.5)	0	0.160
HPeV		3 (1.5)	1(0.8)	0.511
EV		8 (3.9)	8 (6.0)	0.380
HAdV		2 (1.0)	1 (0.7)	0.826
*S*. *aureus*		27(13.1)	13(9.8)	0.400
*H*. *influenzae B*		21 (10.2)	11 (8.2)	0.629
*S*. *pneumoniae*		7(3.4)	2(1.5)	0.282
*L*. *pneumophila/L*. *longbeachae*		1 (0.5)	0	0.418
*M*. *catarrhalis*		7(3.4)	3(2.3)	0.532
*Bordetella spp*.		33(16.0)	26(19.5)	0.365
*H*. *influenzae*		6 (2.9)	2 (1.5)	0.395

The difference among the groups was considered significant when p-value ≤ 0.05.

## Discussion

In the present study we identified the circulating respiratory pathogens in three major cities in Jordan during the winter months December-March 2021/2022. Viral infections among patients with respiratory symptoms were mostly caused by SARS-CoV-2 (39.2%), IBV (17.4%), IAV (10.9%), and EV and HBoV (4.7% each). Bacterial infections were mostly caused by *Bordetella* species, *Staphylococcus aureus*, and *H*. *influenzae B* (17.4%, 11.8%, and 9.4% respectively). *Moraxella catarrhalis*, *S*. *pneumoniae*, *and Haemophilus influenzae* (non-type b) were reported to a lesser extent (2.9%, 2.7%, 2.4% respectively). SARS-CoV-2 was the predominant cause of respiratory infections within the period of sample collection and that was largely affected by the fact that the study period involved two documented surges of COVID-19 cases in the country. The first was around the 6^th^ of December 2021 (confirmed daily cases were around 5,000) just two weeks before starting our sample collection in Al Zarqa city at JZH which explains the dwindling number of positive cases (11/100, 11.0%) reported during that study period [22]. Cases reported during this period were mostly caused by the delta variant as the first omicron cases were recorded in Jordan around mid-December [23, 24]. The second peek was around mid-February 2022 (daily cases were around 22,000) [22], which was the time at which Irbid, and Amman samples were collected at ISH and PHH which justifies the substantial number of positive cases recorded at those locations (30/63, 47.6% and 91/182, 50.0% respectively). The omicron variant during this period was causing 90% of the COVID-19 cases in Jordan [24]. The efficacy of the different COVID-19 vaccines in prevention against SARS-CoV-2 infection was statistically significant (*p* = 0.046). It is noteworthy that our sampling criteria excluded patients with an established diagnosis of SARS-CoV-2 infection at the time of sample collection which suggests that the rates of SARS-CoV-2 infections were likely to be higher during winter 2021/2022.

Our results showed that respiratory viruses other than SARS-CoV-2 were commonly detected in patients presented with URTIs. We found that 76.1% of the 339 tested samples were positive for either single or multiple respiratory pathogens. These results are consistent with earlier reports from different countries with variable age groups. For instance, 73.0% positivity rate was reported from 1,427 URTIS cases in Senegal [25], 78.0% from a total of 334 cases in Indonesia [26], and 78.0% out of 33,404 cases in Qatar [27]. Lower rates were reported in Suriname (45.3% in 1096 individuals) [28] and in Laos (65.1% of 548 subjects) [29]. Several factors play a role in the discrepancy observed among these studies such as the size and demographic make-up of the populations investigated, the length of the study, the virus-detection diagnostic methods, and environmental variables.

Our findings confirm earlier research that the influenza virus is a major contributor to respiratory infections all over the world. During the previous winter 2020/2021, influenza virus infections’ prevalence has recorded small numbers due to the pandemic-associated mitigation measures [30]. The easing of public health restrictions beginning from summer 2021 and into the winter of the same year has led to normal timing of influenza season beginning in Jordan. In this study, we showed that influenza virus predominated among URTI patients, accounting for more than 29.5% of all assessed specimens, these figures are consistent with previous studies where the prevalence of influenza varied from 15.8% to 32.0% [31–35]. Most studies have shown that influenza type A was the most predominant influenza virus type [27, 34, 36, 37], but this study has shown a predominance of type B (n = 59, 59.0%), compared to type A (n = 37, 37.0%), and type C (n = 4, 4.0%), consistent with few other reports [38, 39]. Other respiratory viruses co-circulated with influenza viruses but at lower rates, including HBoV (4.7%), enteroviruses (4.7%), HCoVs (2.4%), HPeV (1.2%), HRSV (0.9%), adenoviruses (0.9%), HRVs (0.6%), and HPIVs (0.6%). These findings are somehow divergent from epidemiological studies conducted on adults in other countries. HBoV viral infection is usually associated with pediatric respiratory illness and rarely seen in adults [40], but in this study it was the third leading cause of URTI after SARS-CoV-2 and influenza. Similar frequency figures for HBoV were seen in other studies but were ranked after RSV, rhinovirus adenovirus, parainfluenza viruses, and coronaviruses [27, 41]. Human enteroviruses were reported at a similar prevalence in previous studies where genotypes A and B [42], or EV-D68 [43] were the most frequent.

Among those positive for CoVs, type 229E was the most prevalent type (n = 6, 75.0%), followed by HKU1 (2, 25%) contrary to most reports where OC43 is usually the most common [44, 45]. Despite being a known causative for URTIs, few studies investigated the epidemiological traits of parechovirus [46]. In the current study, parechovirus infections were recorded in just 1.2% of the cases (n = 4). Low rate of parechovirus infections is expected as these infections are typically more frequent in children than adults [47]. Therefore, it is possible that the low prevalence of parechovirus-associated URTISs in adults is due to the presence of sterilizing immunity, which provides protection and regulates the severity of infection [48]. All other viruses we investigated were observed at a detection rate of less than 1.0%. However, studies that included younger participants reported higher percentages of HRVs and HRSV [49]. Higher HRVs circulation was observed during times of low influenza activity, in line with earlier reports [2]. Subtype analysis of the parainfluenza viruses revealed that subtypes 3 and 2 were the most detected types, which is in accordance with previous studies [50]. Bacterial infections were seen mostly as coinfections rather than single infections where *S*. *aureus*, and *Haemophilus influenzae* (non-type b) were the most common identified bacteria which has little significance as they could colonize the nasopharynx in 30% of the population [51]. On the other hand, higher numbers of *Bordetella* spp. were isolated in agreement with recent reports on the resurgence of pertussis cases and the need of the development of a new generation vaccine [52, 53].

Coinfections were seen with one or more pathogens in 50.4% (67/133) of patients with SARS-CoV-2. Another study that utilized the same detection technique reported coinfection rates that were comparable to those reported in this work [54]. Alternatively, few earlier reports have shown lower rates of respiratory pathogen coinfection with SARS-CoV-2 compared to our study [55, 56]. In patients with SARS-CoV-2, Rho *et al*. showed that 7.9% had viral coinfections and 0.9% had bacterial coinfections [56]. The higher rate of coinfection seen in this study could be attributed to the use of a real-time PCR respiratory panel that detects 33 respiratory pathogen including bacteria, viruses, and a single fungal infection, compared to the use of narrow number of PCR targets in other studies. The higher coinfection rate of 94.2% reported by Zhu *et al*. could once again be attributed to the use of a broad-range respiratory panel testing for 39 respiratory pathogens [57]. Multiple factors could play a role in the variations in the coinfection rates seen in different studies such as: age group of participants, chronic illnesses, and COVID-19 severity of the study’s participants, intake of antibiotics, and lab detection tools [58].

*Bordetella spp*., *S*. *aureus and H*. *influenzae* type B were the most identified bacteria in patients with SARS-CoV-2, which was inconsistent with previous studies. To the best of our knowledge this is the first study that reports *Bordetella spp*. as the most common coinfecting bacteria in COVID-19 patients. Contrary to our study, multiple studies in different countries (Italy, Korea, and India) reported no cases of Bordetella coinfection among COVID-19 patients [54, 56, 59]. These findings need to be interpreted cautiously until further investigations are carried out (ongoing project by our team). Simultaneously, multiple previous reports have reported *S*. *aureus and H*. *influenzae* as common bacterial coinfections [54, 55, 60]. In our study, 14 patients had coinfections with *H*. *influenzae*, including 11 patients with *H*. *influenzae* type b and 3 with non-typeable *H*. *influenzae*. Differentiating whether these are colonizers or true coinfection pathogens may be a tough task and beyond the scope of this study. The weakened cell mediated immunity in COVID-19 patients makes them more susceptible to infection with *Moraxella catarrhalis*, as 4 coinfections with *Moraxella catarrhalis* were reported in this study as well as other studies in patients with COVID-19 [54]. The prevalence of *S*. *pneumoniae* coinfection in COVID-19 patients was 2.7% which is consistent with previous studies [61, 62]. In our study population, only a sole case of atypical bacteria (*Legionella pneumophila*) was found as a coinfection pathogen in COVID-19 patients. There was no evidence of *M*. *pneumoniae*, *K*. *pneumoniae*, *and C*. *pneumoniae* coinfection.

Viral coinfection, however, was seen less frequently compared to bacteria. IBV, HBoV, and enteroviruses were the most common pathogens of viral coinfection. In previously published reports Influenza A virus was seen more frequently as a co-infectious agent with SARS-CoV-2 [63]. In our study, Influenza B virus was reported as a coinfection pathogen in 15 cases compared to zero with influenza A virus. The prevalence and circulation of influenza viruses in Jordan needs to be further studied to draw a robust conclusion from these findings (an ongoing project by our team).

Our study has several limitations. First, sample collection was limited to only three centers in the country. However, since PHH was the primary center for the management of COVID-19 in Jordan, it could have highly reflected a representative sample of patients with URTIs. Moreover, we included two other hospitals in the two other largest cities in Jordan in an effort to improve the power of the patients’ sample. Second, the inclusion of patients from 5–70 years of age and exclusion of children below 5 years of age due to difficulties in obtaining nasopharyngeal swabs from them might have led to lower detection rates of certain respiratory pathogens such as RSV. Lastly, the lack of data regarding the influenza vaccination status of study participants represents another limitation. Recent studies have demonstrated that flu vaccination could lower the risk of flu disease by 40–60% among the general population during flu seasons when the majority of circulating flu viruses are closely matched to those used in the flu vaccines, but these figures drop to 10–20% in case the circulating flu viruses do not match [64]. But since we do not have any data about the use of the flu vaccine by the study participants and still working on identifying the circulating genotypes of the flu viruses during the study period, we cannot confirm whether including a history of influenza vaccination would have affected the number of influenza infections in our study.

## Conclusions

This study is an attempt to determine the prevalence of SARS-CoV-2 and another 33 types of the most frequently described respiratory pathogens in patients with symptoms of URTIs. Since the sample collection period coincided with two COVID-19 surges in Jordan, SARS-CoV-2 was the most prevalent respiratory pathogen detected. Influenza virus was the second most prevalent among viral pathogens, while *Bordetella* species and *Staphylococcus aureus* were the two most prevalent bacterial causes of URTIs. Interestingly, more than 50% of patients with symptoms of URTIs were confirmed to have a coinfection with two or more respiratory pathogens, with SARS-CoV-2 and *Bordetella* coinfection being most predominant. This study, and other similar efforts, might be of great value for the continuous process of monitoring the most common causes of URTIs in the local community.

## Supporting information

S1 Table Correlation between the type of respiratory pathogen and clinical presentation among the study population. (DOCX)

S2 Table Correlation between the type of respiratory pathogen and age, gender, health status, and SARS-CoV-2 vaccination among the study population. (DOCX)
