# Supplementary material for: Prevalence of SARS-COV-2 and other respiratory pathogens among a Jordanian subpopulation during Delta-to-Omicron transition: Winter 2021/2022
Source: PLoS One. 2023 Mar 30;18(3):e0283804. doi: 10.1371/journal.pone.0283804 (PMC10062597; doi:10.1371/journal.pone.0283804)
Supplement: S1 Table — (DOCX) [file pone.0283804.s001.docx]

**S1 Table.** Correlation between the type of respiratory pathogen and clinical presentation among the study population.

| **Symptoms of study population** | | | | | | | | | | | | | | | | | | | | | | | | | | **Characteristic** |
| --- | --- | --- | --- | --- | --- | --- | --- | --- | --- | --- | --- | --- | --- | --- | --- | --- | --- | --- | --- | --- | --- | --- | --- | --- | --- | --- |
| **p-value** | **Nausea** | **p-value** | **Vomiting** | **p-value** | **Diarrhea** | **p-value** | **Dizziness** | **p-value** | **Change of smell or taste** | **p-value** | **Difficulty in breathing** | **p-value** | **Nasal discharge** | **p-value** | **Cough** | **p-value** | **Fever** | **p-value** | **Chills** | **p-value** | **Sore throat** | **p-value** | **Myalgia** | **p-value** | **Headache** |  |
| 0.888 | 26 | 0.8888 | 26 | 0.492 | 11 | 0.160 | 45 | 0.149 | 19 | 0.656 | 44 | **0.004** | 92 | 0.161 | 104 | 0.234 | 74 | **0.014** | 89 | 0.226 | 104 | 0.101 | 96 | 0.059 | 107 | **SARS-CoV-2** |
| 0.354 | 5 | 0.354 | 5 | 0.271 | 1 | 0.678 | 12 | 0.082 | 1 | 0.222 | 19 | 0.735 | 23 | 0.067 | 32 | 0.972 | 19 | 0.651 | 23 | 0.579 | 29 | 0.232 | 28 | 0.911 | 28 | **IAV** |
| 0.633 | 10 | 0.633 | 10 | 0.921 | 4 | 0.090 | 12 | 0.279 | 9 | 0.427 | 23 | **0.022** | 43 | **0.039** | 50 | 0.481 | 28 | 0.443 | 32 | 0.517 | 46 | **0.022** | 32 | 0.466 | 42 | **IBV** |
| 0.766 | 1 | 0.766 | 1 | 0.160 | 1 | 0.366 | 2 | 0.475 | 0 | 0.512 | 2 | 0.527 | 3 | 0.234 | 4 | 0.284 | 1 | 0.505 | 3 | 0.241 | 4 | 0.468 | 2 | 0.997 | 3 | **ICV** |
| 0.875 | 1 | 0.875 | 1 | 0.356 | 1 | 0.835 | 2 | 0.380 | 0 | 0.951 | 2 | 0.629 | 3 | 0.022 | 2 | 0.117 | 5 | 0.038 | 6 | 0.651 | 4 | 0.083 | 6 | 0.632 | 5 | **HCoV 229E** |
| 0.490 | 0 | 0.490 | 0 | 0.695 | 0 | 0.359 | 0 | 0.614 | 0 | 0.303 | 0 | 0.085 | 0 | 0.437 | 1 | 0.963 | 1 | 0.091 | 0 | 0.408 | 2 | 0.043 | 0 | 0.412 | 2 | **HCoV HKU1** |
| 0.267 | 1 | 0.267 | 1 | 0.695 | 0 | 0.359 | 0 | 0.614 | 0 | 0.644 | 1 | 0.782 | 1 | 0.401 | 2 | 0.963 | 1 | 0.234 | 2 | 0.422 | 1 | 0.609 | 1 | 0.014 | 0 | **HRV** |
| 0.626 | 0 | 0.626 | 0 | 0.782 | 0 | 0.122 | 1 | 0.214 | 1 | 0.168 | 1 | 0.410 | 1 | 0.553 | 1 | 0.301 | 0 | 0.401 | 1 | 0.559 | 1 | 0.482 | 1 | 0.562 | 1 | **HPIV-2** |
| 0.626 | 0 | 0.626 | 0 | 0.782 | 0 | 0.517 | 0 | 0.722 | 0 | 0.467 | 0 | 0.410 | 1 | 0.553 | 1 | 0.301 | 0 | 0.401 | 1 | 0.559 | 1 | 0.482 | 1 | 0.083 | 0 | **HPIV-3** |
| 0.179 | 1 | 0.179 | 1 | 0.258 | 0 | 0.200 | 7 | 0.327 | 3 | 0.778 | 5 | 0.320 | 14 | 0.208 | 14 | 0.704 | 9 | 0.403 | 11 | 0.072 | 15 | 0.876 | 11 | 0.550 | 13 | **HBoV** |
| 0.531 | 1 | 0.531 | 1 | 0.075 | 1 | 0.884 | 1 | 0.536 | 0 | 0.207 | 0 | 0.802 | 2 | 0.770 | 2 | 0.600 | 2 | 0.038 | 0 | 0.750 | 2 | 0.991 | 2 | 0.740 | 2 | **HRSV A&B** |
| 0.115 | 2 | 0.115 | 2 | 0.160 | 1 | 0.843 | 1 | 0.475 | 0 | 0.687 | 1 | 0.527 | 3 | 0.965 | 3 | 0.948 | 2 | 0.092 | 4 | 0.255 | 2 | 0.731 | 3 | 0.247 | 2 | **HPeV** |
| 0.965 | 3 | 0.965 | 3 | 0.258 | 0 | 0.472 | 6 | 0.273 | 4 | 0.778 | 5 | 0.198 | 12 | 0.525 | 8 | 0.247 | 6 | 0.469 | 8 | 0.533 | 13 | 0.484 | 12 | 0.994 | 12 | **EV** |
| 0.531 | 1 | 0.531 | 1 | 0.631 | 0 | 0.884 | 1 | 0.222 | 1 | 0.966 | 1 | 0.802 | 2 | 0.770 | 2 | 0.524 | 1 | 0.370 | 1 | 0.631 | 0 | 0.222 | 3 | 0.740 | 2 | **HAdV** |
| 0.319 | 10 | 0.319 | 10 | 0.912 | 3 | 0.301 | 9 | 0.783 | 5 | 0.523 | 12 | 0.331 | 21 | 0.595 | 31 | 0.649 | 22 | 0.613 | 22 | 0.474 | 28 | 0.664 | 28 | 0.123 | 26 | ***S.aureus*** |
| 0.683 | 7 | 0.683 | 7 | 0.848 | 2 | 0.525 | 11 | 0.155 | 6 | 0.445 | 13 | 0.980 | 19 | 0.897 | 24 | 0.847 | 16 | 0.935 | 19 | 0.663 | 25 | 0.866 | 21 | 0.676 | 23 | ***H.influenza B*** |
| 0.139 | 0 | 0.139 | 0 | 0.401 | 0 | 0.628 | 2 | 0.280 | 0 | 0.271 | 0 | 0.104 | 3 | 0.303 | 8 | 0.266 | 3 | 0.379 | 4 | 0.183 | 5 | 0.484 | 7 | 0.174 | 5 | ***S.pneumonia*** |
| 0.626 | 0 | 0.626 | 0 | 0.782 | 0 | 0.517 | 0 | 0.722 | 0 | 0.467 | 0 | 0.224 | 0 | 0.553 | 1 | 0.332 | 1 | 0.232 | 0 | 0.559 | 1 | 0.154 | 0 | 0.562 | 1 | ***L.pneumophila/L.longbeachea*** |
| 0.454 | 1 | 0.454 | 1 | 0.376 | 0 | 0.170 | 1 | 0.371 | 2 | 0.761 | 3 | 0.182 | 8 | 0.243 | 9 | 0.455 | 4 | 0.223 | 4 | 0.733 | 7 | 0.836 | 7 | 0.715 | 7 | ***M.catarrhalis*** |
| 0.909 | 11 | 0.909 | 11 | 0.921 | 4 | 0.852 | 18 | 0.464 | 5 | 0.848 | 21 | 0.964 | 35 | 0.582 | 42 | 0.676 | 29 | 0.691 | 36 | 0.517 | 46 | 0.093 | 34 | 0.793 | 45 | ***Bordetella spp.*** |
| 0.627 | 1 | 0.627 | 1 | 0.429 | 0 | 0.286 | 1 | 0.907 | 1 | 0.292 | 5 | 0.118 | 8 | 0.950 | 6 | 0.181 | 6 | 0.825 | 5 | 0.397 | 7 | 0.302 | 4 | 0.996 | 6 | ***H.influenza*** |
