# Supplementary material for: Prevalence of SARS-COV-2 and other respiratory pathogens among a Jordanian subpopulation during Delta-to-Omicron transition: Winter 2021/2022
Source: PLoS One. 2023 Mar 30;18(3):e0283804. doi: 10.1371/journal.pone.0283804 (PMC10062597; doi:10.1371/journal.pone.0283804)
Supplement: S2 Table — (DOCX) [file pone.0283804.s002.docx]

**S2 Table.** Correlation between the type of respiratory pathogen and age, gender, health status, and SARS-CoV-2 vaccination among the study population.

| **SARS-CoV-2 vaccine** | | | **Health Status** | | | | | **Gender** | | | **Age** | | | | | | **Characteristic** |
| --- | --- | --- | --- | --- | --- | --- | --- | --- | --- | --- | --- | --- | --- | --- | --- | --- | --- |
| **p-value** | **Unvaccinated** | **Vaccinated** | **p-value** | **Other diseases** | **Respiratory diseases** | **Chronic diseases** | **Healthy** | **p-value** | **Female** | **Male** | **p-value** | **>65** | **46-65** | **19-45** | **11-18** | **5-10** |  |
| **0.046** | 36 | 97 | 0.678 | 6 | 9 | 12 | 106 | 0.061 | 62 | 71 | **0.021** | 11 | 50 | 34 | 37 | 1 | **SARS-CoV-2** |
| N/A | 1 | 36 | 0.075 | 0 | 6 | 1 | 30 | **0.001** | 6 | 31 | **0.001** | 1 | 8 | 28 | 0 | 0 | **IAV** |
| N/A | 17 | 42 | 0.815 | 3 | 4 | 5 | 47 | 0.406 | 21 | 38 | 0.112 | 7 | 19 | 14 | 19 | 0 | **IBV** |
| N/A | 2 | 2 | 0.788 | 0 | 0 | 0 | 4 | 0.694 | 2 | 2 | 0.102 | 0 | 0 | 1 | 3 | 0 | **ICV** |
| N/A | 0 | 3 | 0.743 | 0 | 1 | 1 | 4 | 0.042 | 0 | 6 | 0.402 | 1 | 1 | 4 | 0 | 0 | **HCoV 229E** |
| N/A | 0 | 2 | 0.914 | 0 | 0 | 0 | 2 | 0.243 | 0 | 2 | 0.417 | 0 | 0 | 2 | 0 | 0 | **HCoV HKU1** |
| N/A | 0 | 2 | 0.914 | 0 | 0 | 0 | 2 | 0.243 | 0 | 2 | 0.431 | 0 | 2 | 0 | 0 | 0 | **HRV** |
| N/A | 0 | 1 | 0.112 | 0 | 1 | 0 | 0 | 0.410 | 0 | 1 | 0.744 | 0 | 0 | 1 | 0 | 0 | **HPIV-2** |
| N/A | 0 | 1 | 0.967 | 0 | 0 | 0 | 1 | 0.410 | 0 | 1 | 0.744 | 0 | 0 | 1 | 0 | 0 | **HPIV-3** |
| N/A | 3 | 13 | 0.534 | 0 | 1 | 3 | 12 | 0.065 | 10 | 6 | 0.856 | 1 | 7 | 4 | 4 | 0 | **HBoV** |
| N/A | 0 | 3 | 0.215 | 0 | 2 | 1 | 0 | 0.802 | 1 | 2 | 0.700 | 1 | 1 | 1 | 0 | 0 | **HRSV A&B** |
| N/A | 1 | 3 | 0.347 | 2 | 0 | 0 | 2 | 0.694 | 2 | 2 | 0.401 | 1 | 1 | 0 | 2 | 0 | **HPeV** |
| N/A | 4 | 12 | 0.430 | 0 | 2 | 3 | 11 | 0.808 | 6 | 10 | 0.166 | 1 | 10 | 2 | 3 | 0 | **EV** |
| N/A | 0 | 3 | 0.029 | 1 | 0 | 0 | 2 | 0.802 | 1 | 2 | 0.761 | 0 | 2 | 1 | 0 | 0 | **HAdV** |
| N/A | 7 | 33 | 0.246 | 3 | 5 | 3 | 29 | 0.034 | 10 | 30 | 0.852 | 4 | 15 | 11 | 10 | 0 | ***S.aureus*** |
| N/A | 7 | 25 | 0.671 | 2 | 2 | 4 | 24 | 0.724 | 12 | 20 | 0.169 | 2 | 8 | 12 | 9 | 1 | ***H.influenza B*** |
| N/A | 3 | 6 | 0.707 | 0 | 1 | 0 | 8 | 0.661 | 3 | 6 | 0.098 | 2 | 0 | 6 | 1 | 0 | ***S.pneumonia*** |
| N/A | 0 | 1 | 0.112 | 0 | 1 | 0 | 0 | 0.224 | 1 | 0 | 0.744 | 0 | 0 | 1 | 0 | 0 | ***L.pneumophila/L.longbeachea*** |
| N/A | 2 | 8 | 0.728 | 0 | 0 | 1 | 9 | 0.053 | 7 | 3 | 0.190 | 1 | 0 | 6 | 3 | 0 | ***M.catarrhalis*** |
| N/A | 12 | 47 | 0.898 | 2 | 4 | 7 | 46 | 0.357 | 27 | 32 | 0.708 | 5 | 19 | 20 | 14 | 1 | ***Bordetella spp.*** |
| N/A | 5 | 3 | 0.725 | 0 | 1 | 0 | 7 | 0.369 | 2 | 6 | 0.303 | 1 | 1 | 2 | 4 | 0 | ***H.influenza*** |
